# Supplementary material for: Craniocervical Morphometry in Pomeranians—Part II: Associations with Chiari-like Malformation and Syringomyelia
Source: Animals (Basel). 2024 Jun 23;14(13):1859. doi: 10.3390/ani14131859 (PMC11240474; doi:10.3390/ani14131859)
Supplement: Supplementary file 1 [file animals-14-01859-s001.zip › animals-3048796-supplementary.pdf]

animals

Supplementary file

Supplementary Table

Craniocervical Morphometry in Pomeranians—Part II: Associations with Chiari-like Malformation and Syringomyelia

Koen Santifort [1,2,3,*], Sophie Bellekom [2], Ines Carrera [4] and Paul Mandigers [1,3,*]

1 IVC Evidensia Referral Hospital Arnhem, 6825 MB Arnhem, The Netherlands
2 IVC Evidensia Referral Hospital Hart van Brabant, 5144 AM Waalwijk, The Netherlands
3 Expertise Centre of Genetics, Department of Clinical Sciences, Faculty of Veterinary Medicine, Utrecht University, 3584 CS Utrecht, The Netherlands
4 Vet Oracle Teleradiology, Norfolk IP22 4ER, UK
* Correspondence: koen.santifort@evidensia.nl (K.S.); p.j.j.mandigers@uu.nl (P.M.)

Table S1. Contingency table including numbers and percentages (of total) of included dogs' CM and SM classifications.

Classification	SM0	SM1	SM2	Total
**CM0**	20 (20%)	11 (11%)	5 (5%)	36 (36%)
**CM1**	27 (27%)	18 (18%)	13 (13%)	58 (59%)
**CM2**	2 (2%)	3 (3%)	0 (0%)	5 (5%)
**Total**	49 (49%)	32 (32%)	18 (18%)	99 (100%)

CM, Chiari-like malformation; SM, syringomyelia.
